# The pilot experience upon surgical ablation of large liver tumor by microwave system with tissue permittivity feedback control mechanism

**DOI:** 10.1186/1471-2482-14-82

**Published:** 2014-10-22

**Authors:** Po-Chin Liang, Hong-Shiee Lai, Tiffany Ting-Fang Shih, Chih-Horng Wu, Kai-Wen Huang

**Affiliations:** 1 Department of Medical Imaging, National Taiwan University Hospital, Taipei, Taiwan; 2 Institute of Biomedical Engineering, National Taiwan University, Taipei, Taiwan; 3 Department of Surgery, National Taiwan University Hospital, Taipei, Taiwan; 4 Graduate Institute of Clinical Medicine, College of Medicine, National Taiwan University, Taipei, Taiwan; 5 Hepatitis Research Center, National Taiwan University Hospital, Taipei, Taiwan

**Keywords:** Liver cancers, Local tumor ablation, Microwave ablation

## Abstract

**Background:**

Microwave ablation (MWA) is used to treat patients with unresectable liver cancer. Our institution applied a novel microwave generator capable of automatically adjusting energy levels based on feedback related to tissue permittivity. This approach is meant to facilitate ablations over larger areas and provide results of greater predictablility. This paper reports on the safety, efficacy, and feasibility of this new system in the treatment of patients with large liver tumors.

**Methods:**

Between July 2012 and December 2012, a total of 23 patients with malignant liver tumors exceeding 4 cm in diameter underwent surgical MWA using a 902–928 MHz generator. The proposed system used a 14-gauge antenna without internal-cooling. Follow up on tumor recurrence was performed using contrast-enhanced computed tomography or magnetic resonance imaging at 1 month and then at 3 month intervals for a period of at least 12 months following ablation.

**Results:**

Among the cancers treated, 10 were primary hepatocellular carcinomas (HCCs) and 13 were metastatic lesions from primary colorectal cancer (CRLM). The mean tumor size was 5.40 cm (range of 4.0-7.0 cm). A total of 18 patients underwent MWA via open surgery, and 5 received laparoscopic MWA. The mean ablation time was 1982 seconds, with a range of 900-3600 seconds, and the median number of ablation sessions was 2.0 (range of 1–4 sessions). The rate of complete ablation, as defined by a total loss of contrast-enhancement one month post-treatment, was 82.6% (19 of 23 patients), and the rate of local recurrence was 26.3% (5 of 19 patients). For tumors with a diameter of 4.0-7.0 cm, the technical success rate of MWA was higher for HCC patients (70%) than for metastatic liver cancer (53.8%) patients; however, the difference was not statistically significant. All patients survived throughout the observation period, and the morbidity rate was 8.6%.

**Conclusions:**

MWA treatment using the proposed system with tissue permittivity feedback control resulted in a high rate of complete ablation and reduced morbidity. This approach proved to be a fast, easy, and effective option for the ablation of large liver cancers, particularly HCCs.

## Background

Surgical intervention is the gold standard of treatment for liver cancers; however, many patients are unable to undergo liver resection due to a diminished hepatic reservoir, the multifocal nature of tumors, or severe co-morbidities. These challenges led to the development of local tumor ablation as a treatment for small liver tumors which are not amenable to surgical resection [1].

Radiofrequency ablation (RFA) is the most popular local ablative modality due to its efficacy and safety [2,3]. Nonetheless, the high rate of therapeutic failure, particularly for larger tumors, is an issue of concern [3,4]. Another problem is the difficulty in achieving complete tumor ablation near large vessels due to the heat-sink effect, which decreases the efficacy of heating and diminishes the size of the ablation zone [5].

Microwave ablation (MWA) therapy has been widely applied in the treatment of liver and lung tumors [6]. In this procedure, microwave radiation creates a hyperthermic zone by agitating dipole water molecule in tissue, thereby increasing kinetic energy in the area. Recent advances in engineering have facilitated the development of advanced MW devices that are capable of performing larger ablations in a shorter period of time, compared with traditional RF systems [7,8].

Furthermore, the size and shape of the MWA zone is believed to be more consistent and less vulnerable to the heat-sink effect that arises from large vessels adjacent to the lesion [9]. In this way, MWA can overcome a number of limitations of RFA to improve the efficacy of treatment, particularly in the treatment of large tumors.

The newly designed Medwaves AveCure MWA system (San Diego, CA, USA) features a tissue permittivity feedback control mechanism that provides real-time monitoring of ablation conditions as well as the ability to modulate the power and frequency of MW energy [10]. The heating of biologically active tissue can alter the properties of permittivity, such that energy deposition can be maximized and reverse power (a measure of reflectivity), can be minimized. An increase in the amount of forward power leads to an increase in active heating, which is capable of inducing a large ablation zone and eradicating larger tumors [10].

The aim of this study was to evaluate the safety and efficacy of this novel MW system in the treatment of liver cancers exceeding 4 cm.

## Methods

### Patient population

A total of 23 patients with evidence of primary or metastatic liver cancer (ranging 4-7 cm in diameter) underwent MWA between July 2012 and December 2012 at the National Taiwan University Hospital. Diagnoses were established by triphasic computed tomography (CT) or pathologic analysis of specimens. No evidence of vascular invasion or extra-hepatic metastases was observed in these patients during tumor ablation. Complete tumor resection was not feasible in 21 patients, and the resectability of liver cancers was defined simply as tumors that can be resected completely, leaving an adequate liver remnant. The criteria for adequate reservoir means at least 20% of the total estimated liver volume for normal parenchyma, 40% if the liver is injured by chemotherapy or steatosis, or 40%–70% in the presence of liver fibrosis or cirrhosis, depending on the degree of underlying hepatic dysfunction estimated by pre-operative indocyanine green clearance test. There were two patient with large metastatic colon cancers refused to receive liver resection even their liver reservoirs are adequate for liver resection. They both used to suffer from complicated colon operation and hesitate to receive abdominal operation again, so they underwent local ablation. All of the enrolled patients provided informed consent before undergoing the procedure and this study was approved by National Taiwan University Hospital Research Ethics Committee.

### MWA system

This study evaluated the ability of the Medwaves Microwave Generator with a 14 G antenna (straight needle, shaft length of 15 cm with a 4 cm active microwave field radiating proximally from the tip) to deliver MW energy into the target area. The system includes temperature sensors integrated within the probes as well as intelligent permittivity feedback control. This feedback control enables real-time monitoring of ablation conditions as well as the ability to modulate power (10–32 Watts) or frequency (902 MHz – 928 MHz) according to changes in the ablation zone. The feedback control system further maintains the permittivity of tissue and enables tissue to adapt to microwave radiation. This in turn makes it possible to maximize energy deposition and minimize reverse power. Ablation cycles of 600 seconds resulted in ovoid ablation zones exceeding 4.5 cm in diameter. Microwave tumor ablation by this equipment is a standard clinical practice in Taiwan, not sole for the purposes of the study.

### Ablation technique

Patients were selected to undergo surgical MWA either by open surgery or using laparoscopy. The type of procedure was selected according to tumor location, comorbidities, and patient preference. MWA was performed with ultrasound guidance under general anesthesia. A 14-gauge antenna was inserted and positioned at a designated location within the tumors, and the generator was powered in accordance with the manufacturer’s recommendations for sessions of 600 or 900 seconds. In administering MWA, we sought the complete destruction of tumors. This necessitated detailed pre-operative planning regarding the placement of antennae, energy delivery settings, and ablation time. Generally, the tip of the antenna is positioned at the bottom of the tumor margin for the first application. The antenna is then withdrawn in increments of 1-2 cm as multiple, overlapping ablations are performed. Changing the direction of the tip or re-inserting the antenna can also be considered to ensure that the ablation zone covers the entire area of the tumor with a safe margin exceeding 0.5 cm.

During ablation, observation of an expanding hyperechogenic area under sonography was used to judge the shape of the ablation zone. These changes are visible during ablation; however, they tend to diminish more rapidly in MWA than in RFA. Following ablation, the antenna was removed directly without performing a tract ablation procedure.

### Evaluation of therapeutic efficacy

All patients underwent follow-up with contrast-enhanced CT at 1 month post-ablation, and then once every 3 months using cross-sectional contrasted imaging and serum assays of tumor markers. Complete ablation was defined as a uniform loss of contrast enhancement in the area previously occupied by the tumor. Local recurrence was defined as tumor recurrence in the treated area following ablation therapy observed by contrast-enhanced image as enhanced lesions. The technical success of MWA was assessed according to the criteria; complete ablation with no local recurrence during a one-year follow-up. All statistical analyses were performed using SPSS17.0 software. Comparisons were performed using the Fisher exact test for categorical data and paired t-test for quantitative data. A p-value of less than 0.05 was assumed to be statistically significant.

## Results

This study included 15 males and 8 females. Participants had a mean age of 65.8 years (range of 36–82 years), and the follow-up period for every patient was at least 12 months (mean of 16.5 months). Among the 23 liver tumors from the 23 patients, 10 were primary hepatocellular carcinomas (HCCs) and 13 tumors were metastatic lesions from primary colorectal cancer (CRLM). No extra-hepatic malignancy was noted prior to the administration of MWA. Liver function status was classified as Child-Pugh class A in 15 patients (65.2%), Child-Pugh class B in 7 patients (30.5%), and Child-Pugh class C in 1 patient (4.3%). Individual patient demographics are listed in Table 1.

**Table 1:** Patients characteristics

**Variable**	**Value**	**Range**
Age(y), mean ± SD	65.82 ± 12.05	36-82
Gender (male/female), n	15/8	
Albumin (*g/dL*), mean ± SD	3.93 ± 0.61	2.5-5.2
Platelet count (x *10*^*3*^*/*μ*L*), mean ± SD	218.3 ± 108.5	75-592
International normalized ratio	1.01 ± 0.07	0.93-1.16
Aspartate aminotransferase (*IU/L*), mean ± SD	62.13 ± 50.12	19-196
Alanine aminotransferase (*IU/L*), mean ± SD	53.52. ± 62.02	10-267
Total bilirubin (*mg/dL*), mean ± SD	0.61 ± 0.25	0.30-1.13
Etiology, n (*%*)		
Hepatocellular carcinoma	10 (43.5)	
Metastatic colorectal cancer	13 (56.5)	
Child-Pugh score, n (*%*)		
A	14 (65.2)	
B	8 (30.5)	
C	1 (7.1)	

The locations of tumors were evenly distributed on the right and left lobes, with one tumor located within the caudate. The tumors had a mean diameter of 5.41 ± 0.86 cm (range of 4–7 cm). Characteristics of the tumors are listed in Table 2. Among the 23 patients, 5 underwent laparoscopic MWA and 18 underwent open surgical MWA. All procedures were completed without incident. The mean ablation time, defined as the total time the generator was active for ablation, was 1982 ± 687.3 seconds (range of 900-3600 s). The average ablation session was 2.48 ± 0.79 (range of 1-4).

**Table 2:** Characteristics of tumor and MWA procedures

**Variable**	**Value**	**Range**
Tumor size (cm), mean ± SD	5.41 ± 0.86	4-7
MWA procedure, n (*%*)		
Open approach	18 (78.3)	
Laparosopic approach	5 (21.7)	
Ablation session, (n), mean ± SD	2.48 ± 0.79	1-4
Ablation time (sec), mean ± SD	1983 ± 687.3	900-3600

Demographic profiles of HCC and CRLM patients are listed in Table 3. The rate of complete ablation was 82.6% (90% and 76.9% for HCC and CRLM patients, respectively). The local recurrence rate was 26.3% (90% and 84.6% for HCC and CRLM patients, respectively). Technical success was achieved in 14 of the 23 tumors (60.9% overall, or 70% for HCC and 53.8% for CRLM). The rates of complete ablation, local recurrence, and technical success were better for HCC than for CRLM; however, the difference in local recurrence rates was not significant (Table 4). Two of the patients with CRLM and one patient with HCC developed distant recurrence within the follow-up period.

**Table 3:** Comparison of Variables According to cancer type

**Variable**	**HCC (N = 10)**	**Metastatic liver cancer (N = 13)**
Age(y), mean ± SD	61.5 ± 12.5	69.2 ± 9.08
Male sex, n (*%*)	8 (80.0)	7 (53.8)
Child-Pugh score, n (*%*)		
A	5 (50)	9 (69.2)
B	4 (50)	4 (30.8)
C	1 (10)	0 (0)
Tumor location, n (*%*)		
Left lobe	4 (40)	3 (23.1)
Right lobe	5 (50)	10 (76.9)
Caudal lobe	1 (10)	0 (0)
Major vessel attachment, n (*%*)	6 (60)	7 (53.8)
Tumor size (cm), mean ± SD	5.70 ± 1.49	5.31 ± 0.89
MWA procedure, n (*%*)		
Open approach	8 (80)	10 (76.9)
Laparosopic approach	2 (20)	3 (23.1)
Ablation session, n, mean ± SD	2.80 ± 0.79	2.23 ± 0.73
Ablation time (sec), mean ± SD	2190 ± 721.8	1823 ± 641.8

**Table 4:** Comparison of outcome according to cancer type

**Variable**	**HCC (N = 10)**	**Metastatic liver cancer (N = 13)**
Technique success (%)	7 (70.0)	7 (53.8)
Laparoscopic approach (%)	2 (100)	2 (66.7)
Open approach (%)	5 (62.5)	5 (50.0)
Complete ablation	9 (90.0)	10 (76.9)
Laparoscopic approach (%)	2 (100)	3 (100)
Open approach (%)	7 (87.5)	7 (70.0)
Local recurrence	2 (22.3)	3 (30.0)
Laparoscopic approach (%)	0 (0)	1 (33.3)
Open approach (%)	2 (28.6)	2 (28.6)
Hospital stay (d), mean ± SD	5.70 ± 1.49	5.2 ± 1.11
Laparoscopic approach	3.5 ± 0.71	3.67 ± 0.58
Open approach	6.25 ± 1.03	5.86 ± 0.63
Complication, n (%)		
Bile duct dilatation, n (%)	1 (10.0)	1 (7.69)

No immediate complications were recorded during any of the ablation procedures. Two patients did experience mild dilated intrahepatic bile ducts without jaundice-like symptoms; however, no other clinically relevant complications were observed. Additionally, no cases of mortality were noted during the follow-up period. The mean duration of hospital stay for patients who underwent laparoscopic MWA and open surgical MWA was 6.0 ± 0.82 days and 3.6 ± 0.49 days, respectively. This indicates that the laparoscopic approach resulted in shorter hospital stays, compared to open surgery. No difference was observed between HCC and CRLM patients with regard to duration of hospital stays.

## Discussion

RFA has been attracting considerable attention due to the non-invasive nature of the procedure as well as its highly predictable therapeutic efficacy [11,12]. However, for larger tumors, the ablation efficacy of RFA has been less than satisfactory. Specifically, for tumors exceeding 3 cm, the rate of complete ablation is only in the range of 11–25% [13,14]. In recent years, the potential applicability of microwave therapy in the treatment of large liver tumors has become increasingly apparent.

In microwave therapy, a thermal field is created through the emission of ultra-high-speed electromagnetic microwaves from the active tip of the antenna (usually 915 or 2450 MHz). The amount of heat produced in the tissue depends on water content. Specifically, tissue with higher water content absorbs more energy, resulting in the production of more heat. Like RFA, MWA can be performed percutaneously, laparoscopically, or during laparotomy. Percutaneous treatment is less invasive than the other approaches; it can be performed on an outpatient basis and can be repeated to deal with recurrent tumors [15]. Surgical ablation, including laparoscopic and laparotomic approaches, can be applied in locations inaccessible to percutaneous techniques [15]. In addition, the efficacy of ablation is superior to that of percutaneous ablation [16].

Early data has suggested that RFA and MWA techniques have roughly equivalent efficacies with regard to reducing mortality and the ablation of small HCCs [17-20]. In the treatment of larger liver tumors, we hypothesizes that MWA would have an advantage over RFA due to a lower dependence on tissue properties. In addition, MWA is capable of consistently generating higher intra-tumoral temperatures, which accelerate the process of ablation and make it possible to deal with tumors of greater volume [21]. This results in the need for fewer applications per treatment session [22]. The size of the ablation largely depends on microwave frequency and the permittivity of tissue to microwave energy. Two frequencies are used for ablation treatment: 915 mHz and 2450 mHz. The lower frequency has a longer wavelength (approx. 4.3 cm in tissue), which results in a higher direct heating efficiency over a radius of 2.8 cm. In comparison the longer wavelength of 2450 mHz (approx. 2.8 cm) has a direct heating efficiency over a 1.1 cm radius. However, Early MWA systems were unable to respond to feedback at the tissue level (received through the antennae) during the delivery of treatment, which can result in thermal cytotoxic changes at the cellular level that are capable of altering the dielectric properties of the tissue. The amount of energy administered depends on the temperature of the tissue as well as on tissue permittivity. Specifically, decreased permittivity of coagulated tissue (resulting from an increase in tissue temperature) can decrease the efficacy of ablation treatment [10].

Recently, the microwave device and antenna have recently undergone considerable improvements [23,24]. The MWA system by Medwaves AveCure (San Diego, CA, USA) has intelligent permittivity feedback control and probes with unique integrated temperature sensors. These features permit the real-time monitoring of ablation conditions as well as the modulation of MW power and frequency (902 MHz – 928 MHz). The heating of biologically active tissues alters the properties of permittivity. The feedback control system helps to improve the adaptability of tissue to microwave radiation. Thus, energy deposition can be maximized and reverse power can be minimized. Maximizing the amount of delivered forward power helps to increase active heating. This in turn induces cellular death more uniformly throughout the target area. The ability to control the application of microwave energy makes it easier to estimate and control the size of the ablation zone (created by active heat). Moreover, microwave ablation produces intratumoral temperatures that are consistently higher than those achieved using other existing thermo-ablation technologies. Microwave ablation is also effective on tumors of larger volume, and the process can be completed more quickly, even when using a single un-cooled antenna. RFA or MWA systems generally require multiple applicators to create a large ablation zone. Unfortunately, the insertion of multiple applicators inevitably increases the technical difficulty and associated costs of the process, while also increasing the possibility of complications.

In a recent study evaluating MWA with tumor permittivity feedback in lung tissue, Wolf et al. [10] reported ablations adjacent to 4 mm vessels with no evidence of heat sink or collateral vascular damage. Our results, which demonstrated that MWA can lead to improved outcomes compared with conventional forms of thermal ablation, support the findings of Wolf et al.

This is the first study to report on the therapeutic effects of an MWA system with tumor permittivity feedback control for the treatment of large liver tumors. In addition, this study includes a broader cohort than those considered by previous studies.

This study obtained excellent results using MWA with tumor permittivity feedback for the treatment of large liver tumors (>4 cm) using a single antennae (complete tumor necrosis in 19 of 23 treated tumors). Our study dealt with tumors 4-7 cm in diameter, 56.5% of which presented the attachment of hepatic or portal venous trunks. These tumors were found throughout the entire liver at various depths. Tumors with these characteristics are generally difficult to treat using ablation therapy; however, the use of an uncooled-shaft microwave antenna and repeated probe insertions resulted in a high rate of complete ablation (82.6%). The good therapeutic efficacy can be attributed to the novel MWA system and the type surgical approach [25] (78.3% of patients in our study underwent MWA with an open surgical approach). The rate of complete ablation in this study is higher than that previously reported using other thermal ablation modalities such as RFA. Most previous studies attained a complete ablation rate of only 10–25% for large liver tumors treated by RFA [13,14]. Our results were also superior to those of Yin et al. [26] who treated 20 patients with HCC using 2450 MHz MWA system. In that study, the rate of complete ablation was 65% for large (5.0–7.0 cm) liver cancers. However, Xu et al. [27] previously used a multiple electrode insertion technique, which resulted in a higher rate of 86.4% for the complete ablation of tumors 4.0 cm in diameter at 1 month post-ablation.

In the present study, the rates of complete ablation for HCC and CRLM were 90.0% and 76.9%, respectively. Just like other series, the effectiveness of ablation in dealing with CRLM is far lower than when treating HCC [28]. In addition, the long-term outcome of HCC is generally superior to that of CRLM [29].

Evaluating the long-term results and survival rates associated with MWA is beyond the scope of this study; however, it should be noted that the survival rate in this relatively short follow up (12 months) was 100%. This compares favorably with the study of Lu et al. [12], in which the 1-year survival rate for 36 patients was 96%, and that of Dong et al. [30], in which the 1-year survival rate for 185 patients was 92.7%.

MWA appears to have been well-tolerated by our patient group, with no post-operative deaths and few complications related to the ablation procedure. Specifically, none of the patients in this study showed evidence of sepsis, bile duct damage, uncontrollable bleeding, or significant systemic upset. This is in stark contrast with RFA studies, which have reported complications in as many as 33% of patients [31,32]. In addition, Xu et al. [27] reported a complication rate of 9.3% related to MWA among nine patients.

The study has a number of limitations. Primarily, it was a short-term study of limited sample size; therefore, reliable long term results and survival rates could not be properly evaluated. Nonetheless, the current research was only meant to be a preliminary study to investigate the technical efficacy and safety of this new MW technology, according to surgical approach. We did not deal with long-term disease-free survival.

## Conclusions

The current data supports the feasibility of this new MW technology in the treatment of large liver tumors. Intelligent control, real-time feedback, and use of frequencies in the range of 902 and 928 mHz make it possible to maintain tissue permittivity dynamically during the ablation process, thus maximizing the deposition of energy. This approach represents a safe, fast, and efficacious means with which to ablate large tumors. The initial results are encouraging; however, a larger prospective study with a longer follow-up period should be performed to investigate delayed complications and long-term oncologic outcomes related to MWA with tumor permittivity feedback.

## Competing interests

The authors declare that they have no competing interests.

## Authors’ contributions

PCL, CHW and KWH carried out the clinical procedures. PCL and KWH participated in the design of the study and performed the statistical analysis. HSL and TTFS conceived of the study, and participated in its design and coordination and helped to draft the manuscript. All authors read and approved the final manuscript.

## Pre-publication history

The pre-publication history for this paper can be accessed here:

http://www.biomedcentral.com/1471-2482/14/82/prepub
